# Ebola Virus Disease, Democratic Republic of the Congo, 2014

**DOI:** 10.3201/eid2209.160354

**Published:** 2016-09

**Authors:** Carolina Nanclares, Jimmy Kapetshi, Fanshen Lionetto, Olimpia de la Rosa, Jean-Jacques Muyembe Tamfun, Miriam Alia, Gary Kobinger, Andrea Bernasconi

**Affiliations:** Médecins Sans Frontières, Barcelona, Spain (C. Nanclares, F. Lionetto, O. de la Rosa, M. Alia, A. Bernasconi);; Institut National de Recherche Biomédicale, Kinshasha, Democratic Republic of the Congo (J. Kapetshi, J.-J. Muyembe Tamfun);; Public Health Agency of Canada, Winnipeg, Manitoba, Canada (G. Kobinger);; Swiss Tropical and Public Health Institute, Basel, Switzerland (A. Bernasconi); University of Basel, Basel (A. Bernasconi)

**Keywords:** Ebola virus disease, disease outbreaks, Democratic Republic of the Congo, polymerase chain reaction, PCR, viruses

## Abstract

During July–November 2014, the Democratic Republic of the Congo underwent its seventh Ebola virus disease (EVD) outbreak. The etiologic agent was Zaire Ebola virus; 66 cases were reported (overall case-fatality rate 74.2%). Through a retrospective observational study of confirmed EVD in 25 patients admitted to either of 2 Ebola treatment centers, we described clinical features and investigated correlates associated with death. Clinical features were mainly generic. At admission, 76% of patients had >1 gastrointestinal symptom and 28% >1 hemorrhagic symptom. The case-fatality rate in this group was 48% and was higher for female patients (67%). Cox regression analysis correlated death with initial low cycle threshold, indicating high viral load. Cycle threshold was a robust predictor of death, as were fever, hiccups, diarrhea, dyspnea, dehydration, disorientation, hematemesis, bloody feces during hospitalization, and anorexia in recent medical history. Differences from other outbreaks could suggest guidance for optimizing clinical management and disease control.

## Abstract 2

Differences from other outbreaks could suggest guidance for optimizing clinical management and disease control.

Ebola virus disease (EVD), a severe, often fatal illness in humans, has remained a major public health concern in many parts of sub-Saharan Africa since it appeared in 1976 in Zaire (now the Democratic Republic of the Congo [DRC]). The severity of disease caused by Ebola virus (family *Filoviridae*) varies considerably among the 5 known species. Three species (*Zaire ebolavirus* [ZEBOV], *Bundibugyo ebolavirus*, and *Sudan ebolavirus*) have caused outbreaks affecting dozens, and sometimes hundreds, of persons in Africa. Case-fatality rates (CFRs) vary by species and are highest for ZEBOV (up to 90%) (*1*). The largest EVD epidemic occurred during 2014–2016 in West Africa; it resulted in 28,646 cases and 11,323 deaths and affected 10 countries in Africa, Europe, and North America (*2*).

Simultaneously, in 2014, another EVD outbreak occurred in Equateur Province in northwestern DRC. The first case, reported on July 26, 2014, in a pregnant woman married to a bush meat hunter, came from the village of Inkanamongo (*3*,*4*), close to the town of Boende; she was believed to be the index case-patient. This area is ≈700 km from the capital city, Kinshasa. No paved roads connect the 2 settlements, and communications depend mainly on travelers (*3*). The World Health Organization (WHO) declared the outbreak on August 24, 2014 (*4*).

During the outbreak, July 26–October 4, 2014, a total of 66 EVD cases were reported (28 probable, 38 confirmed); 49 deaths were reported, for a CFR of 74%, including deaths in the community of persons with suspected EVD (*5*,*6*). One third of these case-patients were documented to have had direct contact with the index case-patient. A basic reproduction number of 0.84 (*3*) was estimated, which was lower than in any previous EVD epidemic (*7*). In comparison, West Africa had a reproduction number of 1.7–2.0 for the first 9 months of the outbreak (*8*). WHO declared the outbreak in DRC over on November 21, 2014, forty-two days after the last EVD case-patient tested negative and was discharged from the hospital (*5*).

Genome sequencing identified ZEBOV as the outbreak’s causative agent. However, genetic characterization of the virus pointed to a local variant because it has 99.2% of the genome in common with the strain isolated form the 1995 outbreak in Kikwit, DRC, and >96.8% identified in common with the ZEBOV simultaneously circulating in West Africa (*3*,*9*).

To respond to this epidemic, in collaboration with the local authorities, Médecins Sans Frontières (MSF) opened an Ebola treatment center (ETC) to manage persons with suspected or confirmed EVD in Lokolia; this ETC was fully functional on September 10, 2014. A second ETC was set up inside the regional hospital in Boende on August 28. The activities in both ETCs were conducted until week 44. We describe the clinical features of EVD and predictors of death among patients treated in these ETCs during the 2014 outbreak in DRC.

## Methods

We conducted a retrospective observational study on the basis of medical records of all patients with confirmed EVD who were admitted for care at the MSF-supported ETCs in Lokolia and Boende during August 28–November 8, 2014. Patients were identified through the standard case definition established by the Ministry of Health in collaboration with WHO (*3*).

EVD was confirmed by quantitative reverse transcription PCR (RT-PCR), which is considered the most sensitive method and can detect virus from early acute disease through early recovery (*10*). Initially, samples were sent in dry tubes and EDTA to the Institut National de Recherche Biomédicale (Kinshasa, DRC) to be tested; later the Institut National de Recherche Biomédicale set up a field laboratory in the Lokolia ETC. To confirm EVD, samples were sent to the WHO reference center at the Centre International de Recherche Médicales de Franceville in Gabon (*3*). Patients were treated in accordance with then-current protocols established for viral hemorrhagic fever by MSF and WHO urgent interim guidance for case management, endorsed by the Ministry of Health of DRC (*11*,*12*). Patients with confirmed EVD were discharged after a negative RT-PCR result, 3 days without any major symptoms, and general clinical improvement. For a patient suspected to have EVD, discharge required 2 negative PCR results 48 hours apart, of which 1 was performed at least 72 hours after symptom onset.

### Data Collection

We collected epidemiologic and demographic data (age, sex), history of exposure, dates of onset, and symptoms and signs before and during hospitalization on an anonymized encrypted electronic database. Each day, as part of their routine clinical duties in the ETCs, health staff recorded the health condition of patients. At discharge, clinicians reviewed the medical files of each patient and double-checked data at the moment of encoding into Microsoft Excel version 2007 (Microsoft Corp., Redmond, WA, USA) before analysis with Stata version 12 (StataCorp LP, College Station, TX, USA). Fever was defined as an axillary body temperature >37.5°C (>99.5°F). We considered hemorrhagic signs as the following: bloody feces; hematemesis; epistaxis; gingival or oral bleeding; nonmenstrual vaginal bleeding; bleeding after an intramuscular injection or venipuncture; and red eyes/subconjunctival hemorrhage, even though conjunctivitis also can cause this sign (*3*). We grouped as gastrointestinal symptoms the following: nausea, vomiting, diarrhea (bloody and watery diarrhea), and abdominal pain. We measured, in days, the duration of symptoms and signs only for patients who survived. Laboratory data were recorded in a separate register and subsequently were matched with clinical data by using an MSF identification. The cycle threshold (C_t_) result from the first EVD PCR whole-blood test at admission was used as a proxy indicator of ZEBOV viral load (i.e., the lower the C_t_, the higher the viral load) (*13*). An EVD C_t_ of 25 is considered equivalent to a viral load of 1.28 × 10^7^ copies/mL of blood (*14*). C_t_s <20 suggest a very high viral load.

### Statistical Analysis

We restricted our dataset to RT-PCR–positive patients. Results were reported as proportions, means, or medians for the descriptive analysis. We used Fisher exact test to test the hypothesis involving dichotomous variables, Student *t* test to compare 2 parametric continuous variables, and Mood’s median test to compare nonparametric continuous variables. Variables were modeled in a Probit model to test correlation of continuous (C_t_, incubation day, age) or categorical (age group, sex) variables toward the dichotomy outcome (survived/died). Variable normality, when doubtful, was tested with Shapiro-Wilk test to choose the appropriate additional test. The relative risk (RR) and its SE and 95% CI were calculated according to Altman (*15*). Finally, we tested predictors for death through Kaplan-Meier analysis using the log-rank test of equality for categorical variables and Cox proportional hazard regression for continuous variables to calculate the hazard rate. For survival analysis models, we modeled survival from the day of symptom onset with a censoring time of 30 days because all deaths occurred before 30 days after symptom onset. We included the predictor in the model if the test had a p value <0.25. Hypotheses tested were 2-tailed, and we considered statistical significance only in the presence of p values <0.05.

## Results

Sixty-five persons whose illness met the case definition for EVD were admitted to 1 of the 2 MSF-supported ETCs in Boende and Lokolia during outbreak weeks 35–44. For 25 (38%) patients, EVD was confirmed by RT-PCR. The remaining patients with suspected EVD were determined to have malaria or other infections and were treated accordingly. Of the 25 EVD-confirmed patients, 13 (52%) were male. The 25 EVD patients treated at the ETCs represented 66% of the 38 confirmed EVD cases notified during this outbreak. Twelve (48%) of the 25 case-patients died.

The median age of patients with confirmed EVD was 32 years (range 1–77 years) (Table 1). Male patients were older than female patients (p = 0.03): a median of 32 (25th percentile 12, 75th percentile 47) years versus 25 (25th percentile 6.5, 75th percentile 38.5) years, respectively. Case-patients 15–45 years of age were the most represented (11 [44%]); 9 (36%) case-patients were <15 years of age.

**Table 1:** Characteristics of Ebola virus disease case-patients treated in 2 Ebola treatment centers, Lokolia and Boende, Democratic Republic of the Congo, July–November 2014*

Characteristic	Total	Male	Female	p value
Sex, no. (%)	25 (100)	13 (52)	12 (48)	
Median age, y (IQR)	32 (10–39)	32 (12–47)	25 (6.5–38.5)	0.03
Age group, y, no. (%)				
<5	4 (16.0)	1 (7.7)	3 (25.0)	
>5 to <15	5 (20.0)	3 (23.1)	2 (16.7)	
>15 to <45	11 (44.0)	5 (38.4)	6 (50.0)	
>45	5 (20.0)	4 (30.8)	1 (8.3)	
Patient cycle threshold at admission, no. (mean ± SD)	23 (29.22 ± 5.90)	12 (30.61 ± 5.39)	11 (27.7 ± 6.32)	
<5 y	3 (22.07 ± 3.63)			<0.001
>5 to <15 y	5 (35.06 ± 4.64)			
>15 to <45 y	10 (28.17 ± 4.23)			<0.001
>45 y	5 (29.76 ± 6.25)			
Time from onset to admission, mean d ± SD	4.69 ± 2.63	4.61 ± 2.93	4.82 ± 2.34	
Survived	4.25 ± 3.13			
Died	5.21 ± 1.95			
Median hospital stay, d (IQR)	4 (3–12)			
Survived	10 (3–13)	3	24	0.09
Died	4 (2.5–5.5)	1	18	
Time from onset to outcome, d (IQR)	10 (7–15)	3	30	
Survived	11.5 (7.5–19.5)	4	30	0.28
Died	9 (7–12)	3	23	

*Blank cells indicate the numbers were too small for meaningful calculation. IQR, interquartile range.

Time from symptom onset to admission, available for 24 patients, averaged 4.69 days (range 1–9 days). This interval was shorter for survivors (4.25 days) than for persons who died (5.21 days) and for female patients (4.82 days) than for male patients (4.61 days). We did not find any significant difference between the 2 ETCs in terms of delays of hospitalization and outcome. At admission, the most common symptoms reported were asthenia (84%); fever (80%); and anorexia, vomiting, and diarrhea (56% each). In general, gastrointestinal symptoms accounted for 28% of all reported symptoms, but no patients showed signs of dehydration at admission. Hemorrhagic signs, documented at admission for 7 (28%) patients, were mainly gastrointestinal bleedings. Anorexia, myalgia, and abdominal pain occurred significantly more often in adults (patients >15 years of age) than in children (p<0.05). Only anorexia correlated with death (odds ratio [OR] 6.99, p<0.05) (Table 2).

**Table 2:** Symptoms and signs, and their prediction for death, among patients admitted to 2 Ebola treatment centers during the Ebola virus disease outbreak, Lokolia and Boende, Democrtic Republic of the Congo, July–November 2014

Symptom/sign	Survived, no. (%)	Died, no. (%)	Total, no. (%)	Case-fatality rate, %	Relative risk (95% CI)	p value
Symptoms						
Asthenia	10 (76.9)	11 (91.7)	21 (84.0)	52.4	2.09 (0.36–12.00)	0.59
Anorexia	4 (30.8)	10 (83.3)	14 (56.0)	71.4	3.93 (1.07–14.37)	0.01
Headache	5 (38.5)	7 (58.3)	12 (48.0)	58.3	1.52 (0.66–3.5)	0.44
Myalgia	5 (38.5)	6 (50.0)	11 (44.0)	54.5	1.27 (0.56–2.86)	0.69
Right upper quadrant abdominal pain	4 (30.8)	6 (50.0)	10 (40.0)	60.0	1.5 (0.67–3.33)	0.42
Difficulty swallowing	1 (7.7)	4 (33.3)	5 (20.0)	80.0	2 (1–3.9)	0.16
Nausea	2 (15.4)	3 (25,0)	5 (20.0)	60.0	1.33 (0.56–3.16)	0.64
Arthralgia	2 (15.4)	2 (16.6)	4 (16.0)	50.0	1.05 (0.35–3.08)	1
Dyspnea	0	1 (8,3)	1 (4.0)	100.0	2.18 (1.41–3.37)	0.48
Cough	0	1 (8.3)	1 (4.0)	100.0	2.18 (1.41–3.37)	0.48
Back pain	0	1 (8.3)	1 (4.0)	100.0	2.18 (1.41–3.37)	0.48
Disorientation	0	1 (8.3)	1 (4.0)	100.0	2.18 (1.41–3.37)	0.48
Stomach pain/cramps	1 (7.7)	0	1 (4.0)	0		

Signs						
Fever up to 39.5°C	9 (69.2)	10 (83.3)	19 (76.0)	52.6	1.58 (0.47–5.29)	0.64
Vomiting	5 (38.5)	9 (75.0)	14 (56.0)	64.3	2.35 (0.83–6.67)	0.11
Diarrhea	5 (38.5)	9 (75.0)	14 (56.0)	64.3	2.36 (0.83–6.67)	0.11
Fever >39.5°C	1 (7.7)	0	1 (4.0)	0		
Hiccups	0	1 (8.3)	1 (4.0)	100.0	2.18 (1.41–3.37)	0.48
Nonhemorrhagic rash	0	1 (8.3)	1 (4.0)	100.0	2.18 (1.41–3.37)	0.48
Dehydration	0	1 (8.3)	1 (4.0)	100.0	2.18 (1.41–3.37)	0.48

Hemorrhagic signs						
>1 of the signs below	2 (15. 4)	5 (41.7)	7 (28.0)	71.4	1.83 (0.87–3.87)	0.2
Bloody feces	1 (7.7)	4 (33.3)	5 (20.0)	80.0	2 (1–3.9)	0.16
Hematemesis	0	3 (25.0)	3 (12.0)	100.0	2.44 (1.48–4.04)	0.09
Red eyes/subconjunctival hemorrhage	1 (7.7)	1 (8.3)	2 (8.0)	50.0	1 (0.24–4.45)	1
Epistaxis	0	1 (8.3)	1 (4.0)	100.0	2.18 (1.41–3.37)	0.48
Gingival/oral bleeding	0	1 (8.3)	1 (4.0)	100.0	2.18 (1.41–3.37)	0.48
Nonmenstrual vaginal bleeding	0	1 (8.3)	1 (4.0)	100.0	2.18 (1.41–3.37)	0.48

C_t_ at admission was available for 23 (96%) patients. The average C_t_ was 29.2 ± 5.9 (range 17.9–39.1). C_t_ for children <5 years of age was lower, indicating higher viral load, than for children 5–15 years (p<0.01). Two (9%) patients, 1 and 53 years of age, who died had a C_t_ <20. Survivors spent a median of 10 days in the hospital (maximum stay 24 days). The others died a median of 4 days (range 1–18, p = 0.09) after admission.

During hospitalization, asthenia (96%); anorexia and diarrhea (68% each); and vomiting, myalgia, and fever up to 39.5°C (60% each) were the most common symptoms (Table 3). The symptoms of longest duration were asthenia (4 days), red eyes (4 days), tender abdomen (4 days), anorexia (2.9 days), and arthralgia (2.4 days).

**Table 3:** Symptoms and signs developed during hospitalization during the Ebola virus disease outbreak in Ebola treatment centers, Lokolia and Boende, Democratic Republic of the Congo, July–November 2014*

Symptom, sign	Survived, no. (%)	Died, no. (%)	Total, no. (%)	Average no. days	CFR	Relative risk (95% CI)	p value	Patient age group, y, %		p value
								<15	>15	
Symptom										
Asthenia	12 (92.3)	12 (100)	24 (96.0)	4	50.0	0		88.9	100.0	0.36
Anorexia	7 (53.8)	10 (83.3)	17 (68.0)	2.9	58.8	2.35 (0.66–8.33)	0.2	44.4	81.3	0.075
Myalgia	6 (46.1)	9 (75.0)	15 (60.0)	2.3	60.0	2 (0.71–5.62)	0.22	11.1	87.5	0
Arthralgia	5 (38.4)	6 (50.0)	11 (44.0)	2.4	54.5	1.27 (0.56–2.86)	0.69	11.1	62.5	0.017
Headache	6 (46.1)	5 (41.7)	11 (44.0)	2	45.5	0.90 (0.39–2.09)	10	22.2	56.3	0.11
RUQ abdominal pain	3 (23.1)	7 (58.3)	10 (40.0)	2.3	70.0	2.10 (0.92–4.78)	0.11	11.1	56.3	0.034
Difficulty swallowing	2 (15.4)	6 (50.0)	8 (32.0)	1	75.0	2.12 (0.99–4.53)	0.09	0	50.0	0.012
Nausea	3 (23.1)	5 (41.7)	8 (32.0)	2.3	62.5	1.52 (0.69–3.31)	0.41	0	50.0	0.012
Dyspnea	0 (0)	6 (50.0)	6 (24.0)		100.0	3.16 (1.63–6.13)	0.005	11.1	31.3	0.267
Disorientation	0 (0)	6 (50.0)	6 (24.0)		100.0	3.16 (1.63–6.14)	0.005	11.1	31.3	0.267
Cough	1 (7.7)	2 (16.6)	3 (12.0)	1	66.7	1.46 (0.58–3.69)	0.59	0	18.8	0.243
Chest pain	1 (7.7)	2 (16.6)	3 (12.0)	1	66.7	1.46 (0.58–3.69)	0.59	0	18.8	0.243
Back pain	1 (7.7)	1 (8.3)	2 (8.0)	2	50.0	1 (0.24–4.45)	10	0	12.5	0.4
Stomach pain/cramps	2 (15.4)	0	2 (8.0)	1	0	0	0.0	0	12.5	0.4
Diarrhea	6 (46.1)	11 (91.7)	17 (68.0)	2.3	64.7	5.17 (0.8–33.5)	0.03	44.4	81.3	0.075
Fever up to 39.5°C	4 (33.3)	11 (91.7)	15 (60.0)	1.5	73.3	7.33 (1.11–48.2)	0.003	44.4	68.8	0.222
Vomiting	7 (53.8)	8 (66.6)	15 (60.0)	1.3	53.3	1.33 (0.54–3.20)	0.68	100.0	100.0	0.053
Dehydration	1 (7.7)	8 (66.6)	9 (36.0)	1	88.9	3.55 (1.47–8.56)	0.003	22.2	43.8	0.264
Hiccups	0	4 (33.3)	4 (16.0)		100.0	2.62 (1.52–4.53)	0.04	11.1	18.8	0.54
Tender abdomen	1 (7.7)	2 (16.6)	3 (12.0)	4	66.7	1.46 (0.58–3.68)	0.59	0	18.8	0.243
Fever >39.5°C	1 (7.7)	1 (8.3)	2 (8.0)	1	50.0	1.04 (0.24–4.45)	10	11.1	6.3	0.6
Nonhemorrhagic rash	0	1 (8.3)	1 (4.0)	2	100.0	2.18 (1.41–3.37)	0.48	0	6.3	0.64
Hemorrhagic sign										
Hemorrhagic	4 (30.7)	9 (75.0)	13 (52.0)	3.5	69.20	2.77 (0.97–7.87)	0.04	44.4	56.3	0.44
Hemorrhagic, excluding red eyes/subconjunctival hemorrhage	2 (15.4)	9 (75.0)	11 (44.0)	1	85.70	3.81 (1.34–10.8)	0.03	33.3	50.0	0.3
Bloody feces	1 (7.7)	6 (50.0)	7 (28.0)	1	85.7	2.57 (1.25–5.28)	0.03	33.3	25.0	0.499
Bleeding from injection site	1 (7.7)	5 (41.7)	6 (24.0)	1	83.3	2.26 (1.13–4.50)	0.07	22.2	25.0	0.637
Red eyes/subconjunctival hemorrhage	3 (23.1)	3 (25.0)	6 (24.0)	4	50.0	1.05 (0.41–2.67)	10	11.1	31.3	0.267
Hematemesis	0	5 (41.7)	5 (20.0)		100.0	2.87 (1.57–5.19)	0.01	11.1	25.0	0.391
Hemoptysis	0	2 (16.6)	2 (8.0)		100.0	2.30 (1.44–3.66)	0.22	0	12.5	0.4
Epistaxis	0	1 (8.3)	1 (4.0)		100.0	2.18 (1.41–3.37)	0.48	0	6.3	0.64
Gingival/oral bleeding	0	1 (8.3)	1 (4.0)	1	100.0	2.18 (1.41–3.37)	0.48	11.1	0	0.36

*RUQ, right upper quadrant.

In addition to the 7 patients who had hemorrhagic signs at admission, hemorrhagic signs developed in 7 patients during hospitalization. In these patients, bloody feces (7 patients), bleeding from an injection site (6 patients), and red eyes (or subconjunctival hemorrhage) and hematemesis (5 patients) developed. Including red eyes, hemorrhagic signs lasted an average of 3.5 days during hospitalization. Of these 7 patients, 5 died during hospitalization; the 2 survivors had subconjunctival hemorrhage, and 1 had bloody feces. Overall, 14 (56%) patients had hemorrhagic signs during illness. C_t_ at admission was significantly lower for patients with than without hemorrhagic signs (24.83 ± 4.41 vs. 32.59 ± 4.57; p<0.01), and we found no correlation between development of hemorrhage and age or sex.

Nineteen (76%) patients reported at least 1 gastrointestinal symptom before admission. Diarrhea and vomiting were the most common gastrointestinal symptoms (56% each); 10 (40%) patients had both. Ten patients reported abdominal pain. During hospitalization, 2 additional patients had gastrointestinal symptoms, for a total of 21 (84%) patients with at least 1 of these symptoms during illness. Nine (36%) patients also were dehydrated during hospitalization.

Cough was noted for 1 patient, who also had dyspnea, at admission and for 2 patients during their illness. Additionally, at admission, only 1 patient reported dyspnea, and it developed in 5 additional patients during hospitalization (dyspnea developed in 3 patients the day before death). This symptom was suspected to be a preagonic acidosis complication rather than direct respiratory involvement. Moreover, only 1 patient had the characteristic sign of hiccups at admission and it developed in 3 (16%) additional patients during hospitalization. All patients who had hiccups died.

**Figure 1:**
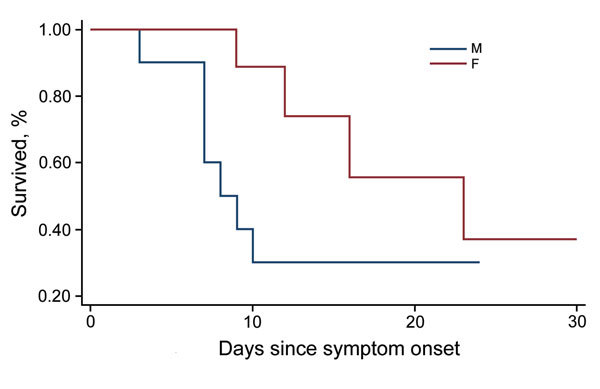
Survival distribution (Kaplain-Meier) by sex of Ebola virus disease patients admitted to 2 Ebola treatment centers, Lokolia and Boende, Democratic Republic of the Congo, July–November 2014.

The CFR was the most important outcome analyzed in this study. Of 25 patients hospitalized with confirmed EVD, 12 died, for an overall CFR of 48%. The CFR was higher for female (67%) than for male (31%) patients (Figure 1). The CFR was highest for children <5 years of age (75%), followed by persons 15–45 years of age (64%). These age groups also had significantly lower average C_t_ (<0.01).

The median time from symptom onset to death was 9 days (25th percentile 7, 75th percentile 12, range 3–23 days). Nine (75%) patients who died had at least 1 hemorrhagic sign during illness, and all hemorrhagic signs except red eyes/subconjunctival hemorrhage were associated with higher CFR. All the 12 patients who died had also vomiting, diarrhea, or both at some point during their illness.

Clinical care for the most severely affected patients focused on maintaining circulatory volume and blood pressure, mainly through maintenance of hydration. Nine patients who died received intravenous treatment.

Symptoms such as fever, hiccups, diarrhea, dyspnea, dehydration, disorientation, hematemesis, and bloody feces that developed during hospitalization significantly correlated with an increased risk for death (p<0.01). Lower C_t_ at admission also correlated significantly with death.

**Figure 2:**
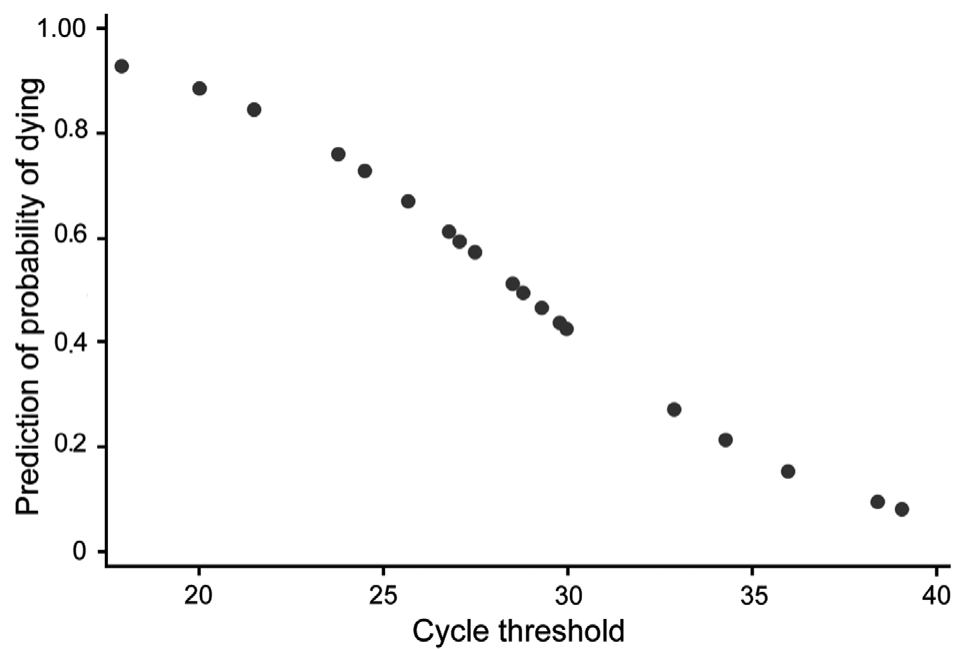
Prediction of Ebola virus disease patients’ probability of dying in relation to their *Zaire ebolavirus* viral load as determined by cycle threshold at admission to an Ebola treatment center, Lokolia and Boende, Democratic Republic of the Congo, July–November 2014.

A Probit regression model that included C_t_ at admission and sex indicated that an increase in C_t_ correlated with a decreased probability of death by −0.038 (95% CI −0.059 to −0.018; p<0.01) (Figure 2). A Cox regression model that included C_t_ at admission, age, and sex confirmed that C_t_ was the best predictor of death (hazard ratio 0.81, 95% CI 0.68–0.96; p = 0.02 (Cox, Table 4).

**Table 4:** Symptoms and signs during hospitalization of Ebola virus disease patients admitted to 2 Ebola treatment centers, Lokolia and Boende, Democratic Republic of the Congo, 2014*

Symptom/sign	Hazard ratio (95% CI)	p value
Fever up to 39.5°C	7.33 (1.11–48.2)	<0.01
Hiccups	2.62 (1.52–4.53)	0.04
Diarrhea	5.17 (0.8–33.5)	0.03
Dyspnea	3.16 (1.63–6.13)	<0.01
Dehydration	3.55 (1.47–8.56)	<0.01
Disorientation	3.16 (1.63–6.14)	<0.01
Hematemesis	2.85 (1.57–5.19)	0.01
Bloody feces	2.57 (1.25–5.28)	0.03
History of anorexia	3.93 (1.07–14.37)	0.01
C_t_ at admission	0.79 (0.64–0.97)	0.03

After multivariate analysis†		
C_t_ at admission	0.81 (0.68–0.96)	0.02
Age, y	1.01 (0.97–1.05)	0.62
Sex, F	4.47 (0.59–10.3)	0.22

*C_t_, cycle threshold. †Cox regression test.

## Discussion

In 2014, in parallel with the large EVD outbreak in West Africa, the DRC faced its seventh outbreak of EVD since the first report of the virus in 1976 (Table 5). By the end of this lesser known outbreak, 49 persons with EVD had died, for a CFR of 74% (*3*), a rate consistent with previous EVD outbreaks in the region (*16*) but higher than with the EVD outbreak caused by Bundibugyo virus in Isiro, DRC, in 2012 (CFR 46.7%) (*17*,*18*) and in Kasai Oriental province, DRC, in 2008 (43.7%) (*19*). Although this rate could be overestimated because of a lack of information about possible survivors in the community, most EVD patients died during the first phase of the outbreak, before the implementation of an adequate referral and treatment system. Following the model established in 1995 (*20*), care for a large number of patients occurred in hospital and the MSF-supported ETCs in Lokolia and Boende. However, during epidemiologic weeks 36–44, the CFR of 48% for the 25 persons with confirmed EVD treated in the 2 ETCs did not differ substantially from the 43% reported by Bah et al. in Conakry, Guinea (*21*), and from the 51% reported by Fitzpatrick et al. in Sierra Leone (*14*).

**Table 5:** Previous Ebola virus disease outbreaks notified in Democratic Republic of the Congo since 1976*

Year	Region	No. cases	No. deaths	Case-fatality rate, %	Species
1976	Yambuku, Mongala district	318	280	88.0	ZEBOV
1977	Tandala, Equateur Province	1	1	100.0	ZEBOV
1995	Kikwit, Bundunu Province	315	254	80.6	ZEBOV
2007	Mweka and Leubo, Kasai Occidental Province	264	187	70.8	ZEBOV
2008–2009	Mweka and Luebo, Kasai Occidental Province	32	14	43.7	ZEBOV
2012	Isiro, Oriental Province	77	36	46.7	BDBV
2014	Boende,Equateur Province	66	49	74.2	ZEBOV

*BDBV, *Bundibugyo ebolavirus*; ZEBOV, *Zaire ebolavirus*.

Although possibly biased by the case definition, asthenia (84%) and fever (80%) were the most common symptoms at admission, followed by diarrhea, anorexia, and vomiting (56% each). Among these, only anorexia correlated with death (p = 0.01). Gastrointestinal symptoms (mainly vomiting and diarrhea) were common at presentation, which numerous other groups also have reported (*8*,*16*,*17*,*21*–*25*). Among all patients in the cohort reported here, at admission at least 7.6 of every 10 patients had 1 gastrointestinal symptom or difficulty swallowing and 4 patients had diarrhea and vomiting, posing an increased risk for transmission, as was documented in West Africa (*8*,*24*,*25*). In contrast, no cholera-like diarrhea symptoms were observed in this outbreak, in contrast to several locations in West Africa during the 2014–2016 outbreak (*26*), and the DRC patients admitted to the MSF ETCs had no clinically relevant sign of dehydration. The high prevalence of gastrointestinal symptoms we noted indicates that these symptoms should not be ignored during EVD screening and as part of transmission control efforts. Failing to recognize gastrointestinal symptoms early during the course of disease increases the potential for fatal misdiagnosis (*16*,*24*,*27*,*28*) and for delayed declaration of the outbreak (*20*,*27*,*29*).

Although the ZEBOV isolated during this outbreak is 99% homologous to the Kikwit strain from 1995 (*3*), the high frequency of maculopapular rash, bilateral conjunctiva injection, and sore throat with odynophagia reported in many of the patients from the 1995 outbreak were not observed in this outbreak (*16*,*30*). Also, high frequencies of coughing and hemoptysis, which were common in the 1995 outbreak, were not observed. We did not find a high prevalence of other symptoms, such as difficulty swallowing, arthralgia, and nausea, among the patients in our study, as was described in Isiro (*17*,*23*) and in Kikwit (*16*). Hiccups, considered a characteristic and severe sign of EVD (*31*), occurred in only 1 patient at admission without the co-presence of any hemorrhagic sign, and hiccups developed in 3 (16%) additional patients during hospitalization, similar to what was described in Kikwit (*16*).

Guimard et al. reported that conjunctivitis was highly predictive of EVD and included it in an algorithm for EVD diagnosis (*20*) because it has been described as a relatively early sign of EVD, detectable in 45%–60% of patients (*30*). At Connaught Hospital (Freetown, Sierra Leone), conjunctivitis was much more prevalent in the early phase of the outbreak in 2014 than in the latter stages (*25*). In our study, the documented sign of red eyes/subconjunctival hemorrhage is mainly attributable to conjunctivitis, and its presence did not increase the risk for death (RR 1.05). By calculating the risk for death in correlation with at least 1 hemorrhagic sign, if we included red eyes/subconjunctival hemorrhage as a hemorrhagic sign, we assessed an RR of 3.81. However, if we excluded conjunctivitis, the RR is 2.77. Although conjunctivitis was considered a risk factor in the early phase of the EVD outbreak in West Africa (*8*) and can be common in EVD patients, our findings suggest its value should be reconsidered.

The prevalence of hemorrhagic signs (52%) among the patients treated during the 2014 outbreak in the DRC did not differ from that described in Uganda (Bundibugyo virus) (*18*,*23*) but was higher than the 41% observed in Kikwit and lower than the 78% in the first EVD outbreak in Zaire in 1976 (*16*,*32*). Recent reports documenting the West Africa epidemic reported lower prevalence: 0.9% (*24*); <1% to 5.7% (*8*); <5% (*26*); 6%, reporting in this case only melena and hematemesis (*25*); and 1.9% (*22*). Only Bah et al. (*21*) reported 51% of EVD patients with hemorrhages, but in this case also subconjunctival redness, which might have been conjunctivitis, was included.

We did not observe a significant correlation between older age and death (p = 0.16) in a Probit model with C_t_ at admission and sex, as described previously (*8*,*18*,*21*,*24*,*33*). Female sex was the main risk factor associated with death (RR 2.16, p = 0.07), but once adjusted for C_t_ and age in a Cox regression model, it was no longer significantly associated with death (hazard rate 4.47, p = 0.22). Other groups have reported that male patients were more at risk than female patients for death in the context of EVD (*33*).

Overall, C_t_ at admission was the only statistically significant predictor of death (p<0.01). Average C_t_ at admission also was lower for case-patients who died than for those who survived (9.5 vs. 11.5, p<0.01). This association between high viral load and death was reported recently (*10*) in West Africa (*14*,*24*) and in Uganda in 2000 (*29*). In our study, low C_t_ also was associated with severe hemorrhagic signs during hospitalization.

We report a clinical dataset for the seventh EVD outbreak in the DRC. Although it comprises all the patients admitted in 2 ETCs, the relatively small number of cases documented is a limitation of the study and should be considered in drawing conclusions from these data. Consequently, some level of selection bias cannot be excluded because some persons with minor symptoms or very serious EVD are likely not to have arrived at an ETC. Nevertheless, our analysis provides a better understanding of symptoms and viral loads in relation to clinical outcome.
